# Iodide of Potassium in the Treatment of Urticaria

**Published:** 1891-05

**Authors:** 


IODIDE OF POTASSIUM IN THE TREATMENT OF URTICARIA.—Stern has successfully treated five cases of chronic urticaria by the administration of iodide of potassium, four of the cases having been rebellious to all the measures usually employed in this disease. The fifth case was one of acute urticaria of a a few days' duration. None of the patients were syphilitic and all were rapidly cured. In one case which had lasted four months the intolerable itching disappeared on the second day of treatment, and a complete cure was obtained after two and a half drachms of the iodide had been administered. In two other cases, one of two years' and the other of six years' duration, the effect of the iodide was equally good, cure following the administration of six and eight drachms respectively.—*London Medical Recorder.*

---

LEUCORRHŒA—By J. D. Elbert, M. D., Dundee, Ind.—

℞. Vaseline, ℥ j.

Golden seal, ʒ j.

Listerine, ʒ j.

Mix all together and stir briskly while being warmed, to reduce the vaseline to a fluid state.

Sig: Use freely on cotton tampon once or twice a day.

—*Ex.*

---

CHRONIC RHEUMATISM—By Prof. Loomis, N. Y. City.—

℞. Kali acetatis, ʒ ij.

Sodii iodidi, ʒ ij.

Magendies solution, ʒ j.

Vinum colchici sem., ʒ ij.

Syrup limonis, ʒ j.

Aq. cinnamomi, ℥ iij.

Sig: ʒ j, four times a day.

—*Ex.*

---

DIARRHŒA OF PUERPERAL SEPTICÆMIA—By Prof. Garrigues.—

℞. Acidi carbolici pur.,
Tinct. iodi. a. a. m xvj.
Muc. acaciæ, ℥ ij.
Aq. dist., ad. ℥ viij.

M. Sig: A tablespoonful every hour.

